# Rheo-Fermentation Dough Properties, Bread-Making Quality and Aroma Characteristics of Red Bean (*Vigna angularis*) Sourdough Induced by LAB *Weissella confusa* QS813 Strain Fermentation

**DOI:** 10.3390/foods12030605

**Published:** 2023-02-01

**Authors:** Chengye Huang, Jing Huang, Binle Zhang, Jacob Ojobi Omedi, Cheng Chen, Liyuan Zhou, Li Liang, Qibo Zou, Jianxian Zheng, Yongqing Zeng, Weining Huang

**Affiliations:** 1 State Key Laboratory of Food Science and Technology, Laboratory of Baking and Fermentation Science, Cereals/Sourdough and Nutritional Functionality Research, School of Food Science and Technology, Jiangnan University, Wuxi 214122, China; 2 Fortune Bakery and Shandong Daoxiangcun Food Industry Co., Ltd., Heze 274000, China; 3 College of Food and Bioengineering, South China University of Technology and Guangzhou Institute of Food Industry, Guangzhou 510000, China

**Keywords:** lactic acid bacteria, sourdough fermentation, exopolysaccharide, red bean bread, volatile aroma compounds

## Abstract

This study investigated the impact of in situ-formed exopolysaccharides (EPS) in red bean (*Vigna angularis*) sourdough fermented by *Weissella confusa* QS813 on dough rheo-fermentation properties, bread-making quality and aroma characteristics of red bean sourdough bread. The EPS formed in red bean sourdough and sourdough-induced acidification improved the maximum dough fermentation height, gas retention coefficient and viscoelastic properties of dough. Doughs had a lower increase rate of total SDS-soluble gluten proteins, a low decline in GMP content and similar free sulfhydryl content to wheat dough. Resultantly, breads showed declines in baking loss and hardness, increase in specific volume and lower moisture loss and staling rate after 7 days of storage. Finally, despite a reduction in the total content of aroma compounds, new aroma compounds such as acetic acid and higher contents of 3-methyl-1-butanol and 2,3-butanediol were enriched in red bean sourdough bread. Sourdough acidification probably promoted interaction of EPS with gluten or red bean proteins through bond interactions to form structures which stabilized gluten in dough and increased water-binding ability in red bean sourdough bread. This study provided a better understanding of the role of EPS in sourdough in improving bread quality and of promising strategies to address consumer demand for nutritious and clean-label products.

## 1. Introduction

Red beans (*Vigna angularis*) are commonly cultivated legumes that have been produced throughout East Asian countries [1]. As a nutrient-dense legume, red beans have been studied for several functional and biological activities [2]; thus, they are considered an economical and sustainable legume in wheat–legume composite flours [3]. However, the utilization of red beans in food products is limited due to antinutritional factors and the presence of beany off-flavors; furthermore, its addition compromises the gluten network and product quality [3].

Sourdough fermentation has been commonly applied to improve several functional, nutritional and sensory attributes of wheat–legume-based bread products [4]. The changes observed were due to lactic acid bacteria (LAB) and/or yeast-related metabolism due to sourdough fermentation, resulting in enzymatic transformation of flour substrates into several functional bioactive volatile (e.g., aroma compounds) and non-volatile (e.g., exopolysaccharides (EPS)) metabolites [5]. Furthermore, to improve quality of wheat–legume products, hydrocolloids have increasingly been used to enhance rheo-fermentation properties of dough [6]. Hydrocolloids can improve doughs by modifying water distribution through their water-binding ability and their ability to interact with gluten, non-gluten proteins and starch components of dough [7]. Compared to chemically modified hydrocolloids, bacterially produced EPS have not been widely used, which could be ascribed to their novel status and limited published studies on their application in baked products [7]. EPS have been introduced into bread either as a purified ingredient or in situ formed in sourdough fermented by EPS-producing LAB strains [8]. Moreover, EPS having linear structures and high molecular weight are more effective in enhancing techno-functional properties of bread [9].

In our earlier study, EPS of high molecular mass and low-branched with 97% α-(1,6) linkages produced by *Weissella confusa* QS813 improved water binding and rheological properties of wheat-based sourdough, resulting in increased loaf volume and softer sourdough steamed breads [8,10]. Furthermore, EPS in red bean sourdough fermented by *Weissella confusa* QS813 improved the gluten and frozen dough quality [11]. However, little is known about the impact of in situ-formed exopolysaccharides in red bean sourdough on resultant dough rheo-fermentation and bread aroma characteristics. Therefore, this study aimed at investigating the impact of EPS formed in red bean sourdough fermented by *Weissella confusa* QS813 on rheo-fermentation properties of wheat dough substituted with 10% red bean sourdough and its resultant bread-making quality. Aroma characteristics of wheat–red bean sourdough bread were also reported by use of Gas Chromatography-Mass Spectrometry analysis.

## 2. Materials and Methods

### 2.1. Materials

Wheat flour and red bean flour were purchased from Peng Tai (Qinhuangdao) Wheat Flour Co., Ltd. (Qinhuangdao, China) and Taizhou Shangmeinuo Food Co., Ltd. (Taizhou, China), respectively. All the chemicals used in this study were of analytical grade.

### 2.2. Red Bean Sourdough Preparation

#### 2.2.1. LAB Strain, Growth Media and Inoculum Preparation

The EPS-producing LAB strain *W. confusa* QS813 was obtained from the culture collection of the Laboratory of Baking Science, Sourdough and Ingredient Functionality Research, Jiangnan University (Wuxi, China). The strain was cultivated at 37 °C for 24 h in MRS medium, then sub-cultured (cell concentration of 10^7^ CFU/mL), centrifuged (6000× *g*, 5 min, 4 °C) and washed twice with sterile water to obtain the LAB cell inoculum.

#### 2.2.2. Red Bean Sourdough Fermentation

To prepare the red bean sourdough enriched with in situ EPS, sucrose (10 %, *w*/*w*) was used to replace red bean flour, then distilled water was added and mixed to produce a dough yield of 250. The LAB inoculum (Section 2.2.1) was then included in the mixture and incubated at 30 °C for 24 h.

#### 2.2.3. Physicochemical Changes in Red Bean Sourdough

The pH, total titratable acidity (TTA) and cell counts of samples were determined in 10 g of sample homogenized in 90 mL of distilled water as described by Omedi et al. [12].

### 2.3. Dough and Bread Preparation

Wheat flour, red bean sourdough, distilled water, salt, butter, sugar, yeast, red bean flour and EPS extracted from red bean sourdough were used as raw materials (Table S1). The dough and bread preparation were carried out as described by Zhang et al. [13], with modifications. All the ingredient (except shortening) were weighed and mixed (Sinmag, Wuxi, China) at low speed (3 min) and high speed (2 min). The shortening was then added and mixed again for 2 min at slow speed and 3 min at fast speed. After mixing, the doughs were shaped and leavened for 90 min at 38 °C, 85 % relative humidity (RH). Baking was performed in an electric oven (Sinmag, Wuxi, China) at 175 °C (top) and 225 °C (bottom) for 25 min, followed by cooling (2 h) at room temperature.

### 2.4. Bread Dough and Bread Analyses

#### 2.4.1. Rheo-Fermentation Properties of Bread Doughs

Rheo-fermentation properties of the doughs were determined as previously described by Tang et al. [8], with modification, using a rheofermentometer F3 (Chopin, Villeneuve-La-Garenne Cedex, France). Tests were performed in triplicate using 150 g dough at 38 °C over a period of 3 h with a 2000 g cylindrical weight. Dough development properties and gaseous release properties of dough were recorded at the end.

#### 2.4.2. Dynamic Rheology Properties of Bread Doughs

Dynamic rheology properties of dough were determined as explained by Li et al. [14] and Tang et al. [8], with modifications. The rheometer used was the discovery hybrid rheometer (DHR-3, TA Instruments, Waters, New Castle, DE, USA) with parallel-plate geometry (20 mm diameter, 1 mm gap) at 25 °C. The dough samples were equilibrated for 5 min and were subjected to an oscillatory frequency sweep test (0.1–10 Hz) with a strain of 0.5. The storage modulus (G′), loss modulus (G″) and loss tangent (tanδ) were measured.

#### 2.4.3. Molecular Weight Distribution of Gluten in Bread Dough by Size-Exclusion High Performance Liquid Chromatography (SE-HPLC)

The molecular weight of gluten proteins in the dough was determined by SE-HPLC [15] equipped with a Shodex Protein column (30 °C) and a UV detector (214 nm) and 0.7 mL/min flow rate.

#### 2.4.4. Sulfhydryl (SH) Content in Bread Doughs

The SH content in dough was determined by Ellman’s reagent (5,5′-dithiobise-2-nitrobenzoic acid, DNTB) as described by Beveridge et al. [16], with modifications. Briefly, 30 mg of freeze-dried dough was suspended in 3 mL of solution (80 mM Tris-glycine buffer, 10 mM EDTA, pH 8.0). The dispersions were oscillated for 30 min at 25 °C at 200 rpm for solubilization, followed by incubation and oscillation in darkness for 30 min after addition of 30 μL of 4 mg/mL DNTB. The samples were then centrifuged at 10,000× *g* for 10 min), followed by absorbance (412 nm) measurement of the supernatant against the blank. SH contents were calculated using the equation: SH (μmol/g) = 73.53 × A _412_ × D/C, where A _412_ was the absorbance value at 412 nm, D was the dilution factor, and C was the sample concentration (mg/mL), respectively.

#### 2.4.5. Baking Loss, Specific Volume and Moisture Content of Bread

Baking loss of bread samples was determined by weight determination of dough before and after baking. Bake loss and specific volume were analyzed upon cooling using a Volscan Profiler (Stable Micro Systems, Godalming, UK); volume was determined by the rapeseed displacement method, and each loaf was weighed. Moisture of bread crumb was determined using the AACC-approved air-oven method (44-15A).

#### 2.4.6. Texture Profile Analysis of Bread

Two bread slices (20 mm thickness) taken from center of each loaf were used to evaluate physical crumb firmness. Texture profile analysis (TPA) was performed as described by Tang et al. [8], with modification, using a texture analyzer (CT3, Brookfield Engineering Laboratory, Middleboro, MA, USA) consisting of a double compression test. Settings used were: test speed of 5 mm/s with a 5 g trigger force to compress the middle of bread crumb to 50 % of its original height.

Hardness (peak force of first compression cycle) was used to describe crumb texture.

Furthermore, changes in hardness reflected as the staling rate of bread was determined at zero and 7 days of storage at room temperature. Rate of staling was calculated using the equation:

$$\mathrm{Rate}\;\mathrm{of}\;\mathrm{staling}=(\frac{\mathrm{crumb}\;\mathrm{hardness}\;\mathrm{of}\;\mathrm{day}\;7-\;\mathrm{crumb}\;\mathrm{hardness}\;\mathrm{of}\;\mathrm{day}\;0}{7})$$

#### 2.4.7. Gas Chromatography–Mass Spectrometry Analysis of Bread

Extraction of aroma compounds in bread samples was done using the solid-phase microextraction (SPME) method. Briefly, a carboxen/polydimethylsiloxiane (CAR/PDMS) coated fibre was conditioned at 250 °C for 1 h prior to volatile extraction. Then, 3 g of sample was weighed into a 20 mL headspace vial for a 40 min volatile extraction analyzed by GC–MS equipped with a column at a 1.0 mL/min flow rate and the GC oven temperature programmed from 40 °C to 100 °C at 6 °C/min and then to 230 °C at 10 °C/min with initial and final holding times of 3 min and 6 min, respectively. The electron-impact mass spectra were generated at 70 eV. The ion source and transfer line temperatures were 200 °C and 250 °C, respectively. Scanning mode was used to detect all compounds in the range of *m*/*z* 33–400 atomic mass units.

#### 2.4.8. Sensory Evaluation of Bread

Sensory evaluation was done on the bread samples using the 9-point hedonic scale to ascertain the overall acceptability. The samples were served to 20 trained panelists (10 males, 10 females, aged between 25 and 35 years) 2 h after baking. The following attributes were evaluated: overall acceptability, color, taste and appearance.

### 2.5. Statistical Analyses

Results of at least three independent assays were presented as mean values. Data was compared using one-way analysis of variance (ANOVA), while multiple comparisons of data was performed by Duncan’s test at *p* < 0.05 level of significance using SPSS version 17.0.

## 3. Results and Discussion

### 3.1. Physicochemical Properties of Red Bean Sourdough

The physicochemical properties of doughs are presented in Table 1. The microbial count of *W. confusa* QS813 after 24 h of red bean sourdough fermentation was 2.98 × 10^8^ CFU/g. Sourdough pH and TTA decreased and increased from 6.3 to 4.34 and 3.68 to 11.07 mL, respectively, after fermentation. The content of lactic acid content was higher than acetic acid content after fermentation by *W. confusa* QS813. In addition, the EPS content was 18.68 g per kg after sourdough fermentation. These results implied that *W. confusa* QS813 successfully synthesized EPS and altered the physicochemical properties of sourdough during fermentation. In sucrose-supplemented sourdoughs, LABs of *Weissella* spp. were found to produce significant levels of EPS during fermentation [17]. Therefore, the in situ-formed EPS in the red bean sourdough was consistent with other published studies. Furthermore, the resulting dough (WR) had lower pH and higher TTA than the other doughs (Table 1).

### 3.2. The Effect of In Situ-Formed EPS in Sourdough on Rheo-Fermentation Properties of Bread Dough

Results of the rheo-fermentation properties of dough are presented in Table 1. Maximum dough fermentation height (Hm, mm) values were 78.70 mm and 69.80 mm for CW and CR, respectively. Compared to CW and CR, Hm values were lowest in AWR, but increased (*p* < 0.05) in WR and EWR. This suggested that chemical acidification reduced fermentation tolerance of the red bean dough due to its weakening effect on the gluten network and dough stability [18]. Furthermore, EPS (WR and EWR) and sourdough (WR) could have formed new linkages with gluten and/or red bean protein-gluten in wheat–red bean dough leading to improved fermentation tolerance and a more stable dough. Total gas produced (V_T_) was highest in CW, then AWR and lowest (*p =* 0.05) in WR, EWR and CR. This could be attributed to yeast activity of doughs during fermentation. As a measure of yeast activity, maximum gaseous release height (H’m, mm) values followed a similar trend to V_T_, where, relative to CW, it increased (*p* < 0.05) in AWR, but decreased in WR, EWR and CR. Similar observations were reported in a recent study [19]. However, compared to CW (88.57), the gas retention coefficient (R, %) was significantly higher in WR and EWR than CR and AWR, an indication that the addition of red bean sourdough with in situ-formed EPS (WR) and extracted EPS (EWR) in wheat–red bean dough stabilized the gluten matrix.

### 3.3. The Effect of In Situ-Formed EPS in Sourdough on Dynamic Rheology Properties of Bread Dough

**Figure 1:**
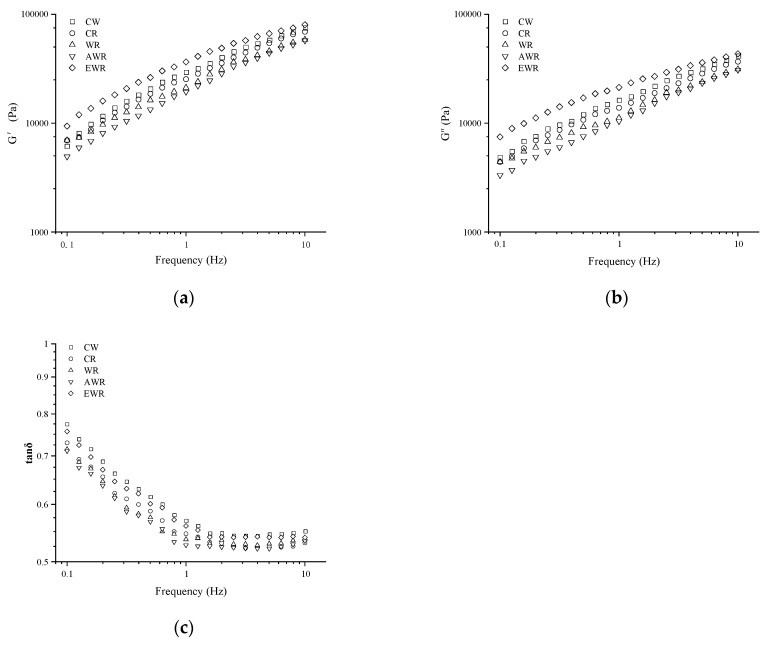
Effect of in situ-formed EPS in sourdough on the rheological properties of dough: (**a**) storage modulus (G’) and (**b**) loss modulus (G″) and (**c**) tan δ of dough.

Changes in the storage modulus (G’), loss modulus (G’’) and loss tangent (tan δ) with an increase in frequency for red bean doughs are presented in Figure 1. Both G’ and G’’ of doughs increased with an increase in frequency, the former being higher than the latter. Compared with CW, G’ and G’’ decreased in CR, WR and AWR, but increased in EWR. This suggested that the addition of extracted EPS increased dough stiffness. This was attributed to the higher water-binding capacity of EPS which competed for available water, leading to incomplete hydration of dough [20]. The rheological properties of doughs acidified by sourdough fermentation (WR) improved when compared with chemical acidification (AWR). This indicated that addition of in situ-formed EPS in sourdough improved dough hydration in WR and alleviated the detrimental effect of chemical acidification (AWR) on the rheological properties of dough. This observation was in agreement with other published studies on the protective role of EPS on the viscoelastic properties of dough [15]. In this study, red bean sourdough in wheat–red bean doughs resulted in dough softening which could be attributed to EPS formation and sourdough acidification.

### 3.4. Size Distribution of Gluten Proteins in Bread Doughs

**Figure 2:**
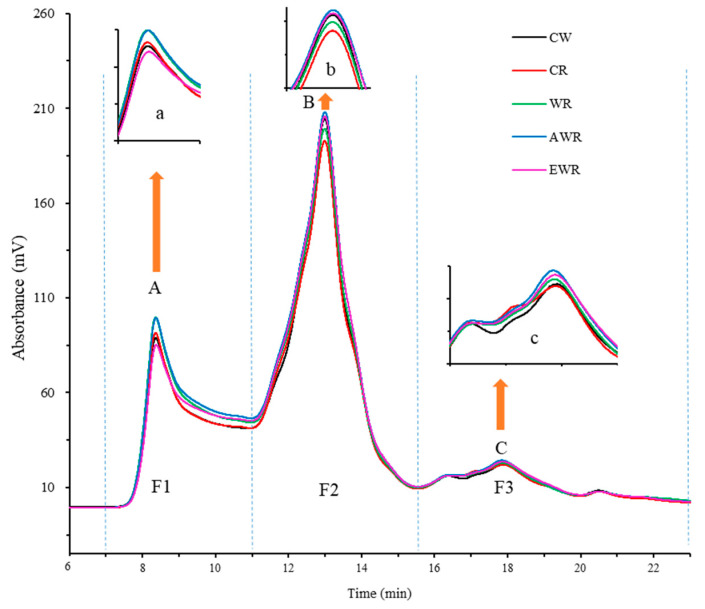
Molecular weight distribution of SDS-soluble proteins from red bean dough analyzed by SE-HPLC. Fractions: polymers (91–688 kDa, F1), monomers (10–91 kDa, F2) and peptides and amino acids (under 10 kDa, F3). Where, A (a), B (b), and C (c) represented peak separations at different magnification for dough samples.

The effect of substituting 10% of wheat flour with red bean sourdough containing EPS on gluten protein size distribution in the dough system analyzed by SE-HPLC is presented in Figure 2. Peaks in the SE-HPLC profiles were divided into three main fractions, namely F1: glutenin polymers (91–688 kDa), F2: monomeric proteins (10–91 kDa) and F3: peptides and amino acids (<10 kDa). The distribution of the gluten protein molecular weight fractions in the doughs were different. The fraction content of the protein in the doughs presented in Table 2 showed that, compared with CW, the total SDS-soluble gluten proteins (PP: F1 and MP: F2 and F3) increased; however, the lowest rate of increase was seen in sourdoughs containing in situ-formed EPS (WR) and extracted EPS (EWR) while the highest was in AWR, followed by CR. The changes observed were indicative of the degree of non-soluble glutenin macropolymer (GMP) depolymerization, which may have degraded GMP and generated smaller gluten proteins [21]. Compared with CW, GMP decreased (*p* < 0.05) from −18.80 % to -32.17 %, with lower declines observed in WR and EWR than CR and AWR. Increased acidification could have increased unfolding and solubility of gluten proteins in dough [19]. Furthermore, red bean flour may have altered the gliadin/glutenin ratio of gluten proteins in wheat dough which would be functionally detrimental and negatively alter the viscoelastic network formation [22]. As a result, compared with CW, proteins in red bean increased total PP content, but were depolymerized into smaller protein units which increased MP content in doughs, especially in CR and AWR. This suggested that presence of extracted EPS (EWR) and in situ-formed EPS in sourdough (WR) improved the gluten network in sourdough-supplemented dough, which enhanced its ability to retain gas-produced proofing. However, chemical acidification and addition of red bean flour weakened the gluten network and lowered the dough’s ability to retain gas produced during proofing. Changes in gluten molecular weight distribution in dough samples were consistent with findings on rheo-fermentation properties of dough (Table 1).

### 3.5. Free SH Content in Bread Dough

The SH content in dough is presented in Table 2. The SH content in doughs were in the range 5.02 to 5.16 µmol/g, with lower and highest content observed in EWR and AWR, respectively. This implied that chemical acidification increased the disulfide (SS) bond disruption of gluten in dough [18]. Similar observations were reported by Su et al. [19], who found that organic acids had a disruptive effect on SS bonds of gluten which increased the SH content. On the other hand, EPS significantly lowered SS bond disruption, probably by forming linkages with wheat–red bean dough components [20]. Relative to EWR and AWR, significantly higher and lower SH contents were observed in controls (CW, CR) and WR, respectively (Table 2). This suggested that sourdough acidification promoted the interaction of EPS with SS bonds of gluten proteins and/or proteins from red bean in the wheat–red bean dough matrix, thereby preserving gluten integrity in dough [6]. In this study, the changes in SH were consistent with findings on the viscoelastic characteristics of the doughs (Figure 1).

### 3.6. Quality Characteristics of Red Bean Sourdough Bread

Quality characteristics of red bean breads prepared with EPS formed in red bean sourdough are presented in Table 3. Compared with control wheat bread (CW), baking loss significantly declined by −3.28 in CR, but increased in WR (1.33), EWR (0.7) and AWR (2.54). Addition of sourdough can weaken the gluten protein network, subsequently increasing baking loss of bread [23]. Relative to EWR and AWR, changes in baking loss in WR implied that in situ-formed EPS in sourdough mildly reduced the weakening effect of acidification on gluten in bread. This was consistent with observations made regarding changes in the molecular weight distribution of gluten, SH content and rheo-fermentation properties of red bean dough containing EPS formed in red bean sourdough. Specific volume of breads revealed that compared with CW, values significantly declined in CR. Specific volume was similar in WR, EWR and AWR, but comparatively lower and higher in CW and CR, respectively. In a study by [19], incorporation of organic acids at proper concentrations was found to improve the specific volume of bread, which was attributed to reduced density and intensity of the gluten network and decreased resistance to dough expansion during baking. Despite having the lowest gas retention coefficient (Table 1), AWR had statistically similar specific volumes as WR and EWR, which suggested that dough acidification was the primary factor that enhanced the specific volume of bread in this study.

Moisture content and hardness of fresh breads (0 days) were different (*p* < 0.05) (Table 3). Compared with CW, moisture content increased in all breads, with WR showing the highest increase, followed by CR, AWR and EWR, which had similar (*p =* 0.05) content. For hardness, lowest (*p* < 0.05) values were observed in WR and EWR bread samples, followed by AWR, and higher (*p =* 0.05) values in CR and CW. This suggested that EPS and, to a lesser extent, acidification, significantly affected amylose retrogradation and moisture migration during cooling of bread. Compared with a study by [24], who reported a neutralizing effect of acidification on EPS of different molecular weights on the firmness and moisture content of fresh wheat sourdough bread, findings in our study suggested that acidification due to sourdough addition could have promoted EPS functionality as a hydrocolloid and interaction with starch components; this would have increased water binding in red bean bread, thereby, slowing down moisture migration and loss in bread. Resultantly, after seven days of storage, moisture loss and staling rate of bread was lowest in WR and EWR, then AWR and CW, and highest in CR (Table 3). This suggested that sourdough acidification and EPS-containing sourdough altered and improved the water-holding properties of bread, which reduced water migration and the staling rate in bread during storage [25]. Subsequently, moisture loss and the staling rate of red bean bread reduced during storage.

### 3.7. The Aroma Compounds in Red Bean Sourdough Bread Crumb

Forty-four volatile compounds, including nine acids, nine alcohols, ten esters, four aldehydes, one ketone, seven hydrocarbons and four heterocyclic compounds were detected in bread crumbs (Table 4). Based on the ANOVA test of total peak area of volatile compoundst of bread, significant decreases relative to CW were observed in all breads in the order WR < EWR < AWR <CR. An increased number of volatile compounds was observed in WR (34), AWR (36), EWR (36) and CR (34) compared with CW (27 types). Relative to CR, total volatile aroma content increased the most (*p* < 0.05) in WR (12.36), followed by EWR (9.14) and AWR (3.93). The findings in this study were in agreement with other published studies which reported that the addition of sourdough reduced total aroma compound content, but increased other types of aroma compounds in bread crumbs [26,27]. Alcohols (e.g., ethanol, phenethyl alcohol, 2-methyl-1-propanol, 2-methyl-1-butanol, benzyl alcohol and 2-3-butanediol) and acids (e.g., octanoic acid, nonanoic acid, oxalic acid and hexanoic acid) were the dominant aroma compounds in crumbs. Compared with CW, alcohol and acid contents decreased and increased, respectively, in WR, AWR and EWR, but both decreased in CR. The aroma compounds may have originated from metabolic activities of yeast during the dough and bread preparation [26,27]. Interestingly, acetic acid was not detected in CW and was highest in EWR then CR, AWR, and least in WR. This suggested that EPS formed in sourdough and sourdough acidification of wheat–red bean dough impacted bread aroma.

### 3.8. Principal Component Analysis (PCA) of Aroma Compounds of Red Bean Sourdough Bread Crumb

**Figure 3:**
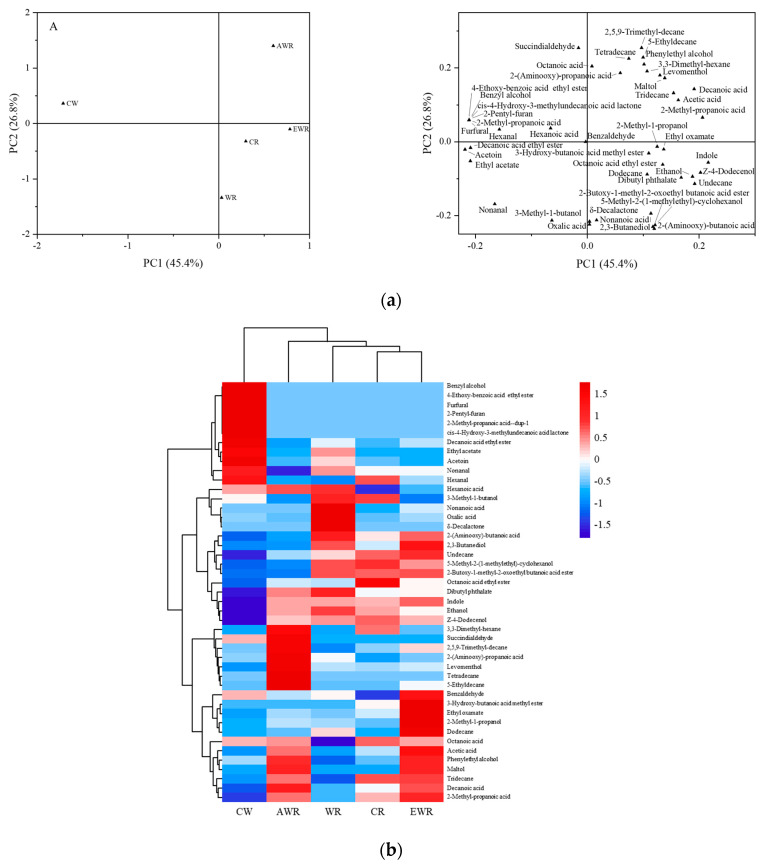
(**a**) Principal component analysis of volatile aroma compounds of wheat–red bean sourdough bread. (**b**) Effect of in situ-formed EPS on volatile compounds of red bean sourdough bread. The color code indicates relative abundance, ranging from blue (low abundance) to white to red (high abundance).

PCA and cluster analysis using heatmaps were used to further understand the effects of EPS in red bean sourdough on aroma compounds of bread crumbs. PCA plots and heatmaps are presented in Figure 3a,b, respectively. The variance of principal components (PC1 and PC2) was 72.2 and loading plots showed clear separations (Figure 3a). This suggested that the volatile composition patterns of breads were different. In PCA plots (Figure 3a) and heatmap (Figure 3b), less ethanol, Z-4-dodecenol, undecane, dibutyl phthalate and indole were observed in CW compared with other breads. However, benzyl alcohol, 4-ethoxy-benzoic acid ethyl ester, decanoic acid ethyl ester, furfural and 2-methyl-propanoic acid were higher in CW than in tother breads. As a result, CW was distantly located from other breads (Figure 3a(A)). Furthermore, the loading plot (Figure 3a(B)) showed that AWR had a higher content of levomenthol, 2-(aminooxy)-propanoic acid, hydrocarbons, maltol and decanoic acid. Higher concentrations of 3-methyl-1-butanol, 5-methyl-2-(1-methylethyl)-cyclohexanol, 2,3-butanediol and Z-4-dodecenol were observed in CR, WR and EWR. These results suggested that the addition of red bean sourdough changed the composition of aroma compounds in wheat–red bean bread [4].

### 3.9. Sensory Evaluation of Bread

The sensory evaluation results of bread are presented in Table 5. Bread prepared with raw red bean flour (CR) had the lowest score on appearance and overall acceptance, while wheat bread (CW) had the best taste. Compared with control wheat bread (CW), the appearance and overall acceptance was higher in WR and lower in AWR and EWR, respectively. Therefore, in this study, the sensory quality was considered highest in WR, followed by CW, EWR, and AWR, and least in CR. The observations made on the sensory analysis could be due to the effect of sourdough fermentation, in situ-formed EPS and acidification which synergistically interact with components in dough to enhance the sensory acceptance of bread [28].

## 4. Conclusions

In conclusion, red bean sourdough containing EPS synthesized by *Weissella confusa* QS813 improved the trheo-fermentation and viscoelastic properties of red bean doughs. Changes in the molecular weight distribution and SH content of gluten in doughs containing in situ-formed EPS in red bean sourdough suggested that sourdough acidification promoted interaction of in situ-formed EPS with gluten proteins and/or red bean proteins which improved the gluten network in red bean dough. These changes improved the gas retention capacity of red bean dough containing EPS formed in red bean sourdough relative to dough containing red bean flour, chemically acidified and extracted EPS in red bean doughs. Finally, bread-making quality, aroma characteristics and overall acceptability of bread prepared with in situ-formed EPS in red bean sourdough were significantly improved as acidification due to sourdough addition promoted EPS functionality, which increased water binding ability in red bean bread. This study presents a better understanding of the potential role played by in situ-formed EPS in red bean sourdough fermented by *W. confusa* QS813 in bread quality improvement of red bean composite dough systems and their use as a promising strategy for value addition to promote legumes such as red bean in the bakery food industry.

## Figures and Tables

**Table 1:** Physicochemical properties of red bean sourdough and its effect on dough development and gas production of the resultant dough and their respective controls.

								Red Bean Dough					
Physicochemical Parameters of Sourdough								Physicochemical Properties		Dough Development Properties		Gaseous Release Properties	
pH	TTA (mL)	LAB (CFU/g)	EPS (g/kg Sourdough)	Lactic Acid (mmol/100 g)	Acetic Acid (mmol/100 g)	FQ	Sample Name	pH	TTA (mL)	H_m_ (mm)	H’m (mm)	V_T_ (mL)	R (%)
4.34 ± 0.05	11.07 ± 0.15	2.98×10^8^	18.68 ± 0.15	106.97 ± 0.16	62.66 ± 0.21	1.71	WR	5.31 ± 0.05 ^a^	4.66 ± 0.11 ^c^	68.40 ± 0.22 ^b^	68.30 ± 0.21 ^a^	1464.00 ± 1.90 ^a^	87.16 ± 0.18 ^c^
Nd	Nd	Nd	Nd	Nd	Nd	Nd	CW	5.48 ± 0.08 ^b^	3.68 ± 0.04 ^a^	78.70 ± 0.24 ^d^	73.10 ± 0.15 ^c^	1540.00 ± 3.00 ^c^	88.57 ± 0.11 ^d^
Nd	Nd	Nd	Nd	Nd	Nd	Nd	CR	5.46 ± 0.02 ^b^	4.05 ± 0.06 ^b^	69.80 ± 0.09 ^c^	71.70 ± 0.08 ^b^	1486.00 ± 4.00 ^a^	84.32 ± 0.08 ^b^
Nd	Nd	Nd	Nd	Nd	Nd	Nd	EWR	5.42 ± 0.04 ^b^	4.01 ± 0.05 ^b^	68.70 ± 0.18 ^b^	68.50 ± 0.18 ^a^	1473.00 ± 3.20 ^a^	87.44 ± 0.25 ^c^
Nd	Nd	Nd	Nd	Nd	Nd	Nd	AWR	5.33 ± 0.07 ^a^	4.61 ± 0.09 ^c^	67.00 ± 0.15 ^a^	75.10 ± 0.22 ^d^	1525.00 ± 2.25 ^b^	87.44 ± 0.25 ^c^

Means ± standard deviation (*n* = 3); different small superscripts in the same column indicate significant differences at *p* < 0.05. H_m_: maximum dough fermentation height; H’m: maximum gaseous release height; V_T_: total gas volume produced; and R: gas retention coefficient (percentage of total gas produced that was retained). FQ: Fermentation quotient. Nd: Not determined.

**Table 2:** The effect of EPS formed in red bean sourdough on molecular weight distribution of gluten proteins and free SH content of wheat–red bean dough.

		Molecular Weight Distribution (%)			
Samples	Free SH Content (μmol/g)	PP	MP	GMP	PP:MP Ratio
CW	5.11 ± 0.05 ^bc^	21.01 ± 0.18 ^a^	47.87 ± 0.08 ^a^	31.12 ± 0.18 ^e^	0.4389
CR	5.08 ± 0.04 ^bc^	23.94 ± 0.11 ^b^	53.05 ± 0.05 ^d^	23.01 ± 0.18 ^b^	0.4513
WR	5.12 ± 0.06 ^bc^	24.45 ± 0.02 ^c^	50.74 ± 0.18 ^c^	24.81 ± 0.08 ^c^	0.4819
AWR	5.16 ± 0.03 ^c^	25.96 ± 0.18 ^d^	52.93 ± 0.15 ^d^	21.11 ± 0.05 ^a^	0.4905
EWR	5.02 ± 0.01 ^a^	24.28 ± 0.05 ^c^	50.45 ± 0.11 ^b^	25.27 ± 0.18 ^d^	0.4813

Means ± standard deviation (*n* = 3); different small superscripts in the same row and column, respectively, indicate significant differences at *p* < 0.05. PP: Polymeric proteins. MP: Monomeric proteins. GMP: Glutenin macropolymers. SH: Sulfhydryl.

**Table 3:** Effects of in situ-formed EPS on baking characteristics of red bean sourdough bread.

Samples	Baking Loss (%)	Specific Volume (mL/g)	Moisture (%), 0 day	Moisture Loss (%/day), 7 days	Hardness (g), 0 day	Staling Rate (g/day), 7 days
CW	27.12 ± 0.11 ^b^	8.44 ± 0.05 ^c^	37.48 ± 0.22 ^a^	0.78 ± 0.01 ^b^	300.33 ± 2.21 ^c^	173.76 ± 1.35 ^b^
CR	26.23 ± 0.06 ^a^	7.01 ± 0.09 ^a^	38.84 ± 0.13 ^b^	0.81 ± 0.02 ^b^	302.33 ± 1.64 ^c^	191.33 ± 4.58 ^c^
WR	27.48 ± 0.12 ^cd^	8.04 ± 0.06 ^b^	39.28 ± 0.06 ^c^	0.73 ± 0.01 ^a^	243.00 ± 5.12 ^a^	136.95 ± 0.79 ^a^
AWR	27.81 ± 0.29 ^d^	8.16 ± 0.09 ^b^	38.91 ± 0.08 ^b^	0.77 ± 0.01 ^b^	275.00 ± 3.24 ^b^	137.71 ± 1.37 ^a^
EWR	27.31 ± 0.13 ^bc^	8.01 ± 0.14 ^b^	38.86 ± 0.05 ^b^	0.73 ± 0.02 ^a^	248.00 ± 4.85 ^a^	135.43 ± 0.81 ^a^

Means ± standard deviation (*n* = 3); different small superscripts in the same row and column, respectively, indicate significant differences at *p* < 0.05.

**Table 4:** Volatile compounds of red bean sourdough bread and the respective controls.

		Peak Area (×10^6^)				
Type	Compounds	CW	CR	WR	AWR	EWR
Acids	Decanoic acid	Nd	6.12 ± 0.06 ^b^	3.24 ± 0.03 ^a^	12.2 ± 0.1 ^d^	9.80 ± 0.08 ^c^
	Octanoic acid	15.2 ± 0.2 ^b^	17.5 ± 0.3 ^c^	1.84 ± 0.04 ^a^	16.0 ± 0.5 ^b^	15.6 ± 0.3 ^b^
	Nonanoic acid	11.2 ± 0.4 ^b^	7.82 ± 0.11 ^a^	55.3 ± 0.5 ^d^	11.8 ± 0.2 ^b^	17.8 ± 0.05 ^c^
	Hexanoic acid	36.3 ± 0.3 ^c^	26.8 ± 0.3 ^a^	38.7 ± 0.4 ^d^	37.9 ± 0.6 ^d^	30.8 ± 0.2 ^b^
	Acetic acid	nd	25.6 ± 0.2 ^b^	2.57 ± 0.07 ^a^	57.7 ± 0.5 ^c^	89.3 ± 0.9 ^d^
	Oxalic acid	7.04 ± 0.14 ^c^	6.08 ± 0.12 ^b^	33.5 ± 0.04 ^e^	5.64 ± 0.08 ^a^	8.47 ± 0.15 ^d^
	2-Methyl-propanoic acid	94.7 ± 0.9 ^e^	36.3 ± 0.4 ^b^	17.0 ± 0.3 ^a^	41.7 ± 0.3 ^c^	50.6 ± 0.5 ^d^
	2-(Aminooxy)-propanoic acid	2.35 ± 0.04 ^c^	0.82 ± 0.02 ^a^	3.71 ± 0.09 ^d^	9.92 ± 0.08 ^e^	1.98 ± 0.04 ^b^
	2-(Aminooxy)-butanoic acid	2.90 ± 0.02 ^a^	15.7 ± 0.2 ^c^	27.1 ± 0.4 ^e^	6.89 ± 0.05 ^b^	21.8 ± 0.5 ^d^
	Sub-total	170 ± 3 ^b^	143 ± 2 ^a^	183 ± 3 ^c^	200 ± 4 ^d^	246 ± 3 ^e^
Alcohols	3-Methyl-1-butanol	45.1 ± 0.5 ^c^	77.3 ± 0.8 ^d^	87.6 ± 0.9 ^e^	8.38 ± 0.08 ^b^	2.74 ± 0.03 ^a^
	2-Methyl-1-propanol	8.28 ± 0.06 ^a^	12.9 ± 0.1 ^b^	20.2 ± 0.2 ^c^	22.3 ± 0.3 ^d^	81.9 ± 0.8 ^e^
	Ethanol	26.2 ± 0.3 ^a^	376 ± 4 ^c^	441 ± 5 ^d^	373 ± 4 ^c^	318 ± 3 ^b^
	5-Methyl-2-(1-methylethyl)-cyclohexanol	nd	58.2 ± 0.6 ^d^	54.8 ± 0.6 ^c^	7.50 ± 0.12 ^a^	47.1 ± 0.5 ^b^
	Phenylethyl alcohol	208 ± 5 ^a^	205 ± 3 ^a^	195 ± 6 ^a^	229 ± 3 ^b^	230 ± 5 ^b^
	Benzyl alcohol	862 ± 9 ^d^	24.3 ± 0.3 ^a^	24.7 ± 0.2 ^a^	29.0 ± 0.3 ^b^	33.5 ± 0.5 ^c^
	Z-4-Dodecenol	nd	37.5 ± 0.5 ^c^	34.6 ± 0.3 ^b^	31.4 ± 0.6 ^a^	31.9 ± 0.2 ^a^
	Levomenthol	nd	14.6 ± 0.3 ^a^	17.3 ± 0.5 ^b^	65.9 ± 0.6 ^c^	18.1 ± 0.3 ^b^
	2,3-Butanediol	47.3 ± 0.3 ^a^	54.4 ± 0.5 ^b^	63.6 ± 0.6 ^c^	47.7 ± 0.3 ^a^	68.8 ± 0.8 ^d^
	Sub-total	1200 ± 16 ^e^	861 ± 10 ^c^	939 ± 11 ^d^	814 ± 8 ^a^	832 ± 10 ^b^
Aldehydes	Nonanal	28.8 ± 0.3 ^c^	15.6 ± 0.2 ^a^	21.3 ± 0.6 ^b^	nd	15.7 ± 0.3 ^a^
	Hexanal	5.43 ± 0.05 ^e^	4.23 ± 0.04 ^d^	0.80 ± 0.06 ^a^	1.15 ± 0.02 ^b^	2.04 ± 0.01 ^c^
	Succindialdehyde	6.42 ± 0.07 ^a^	nd	nd	15.2 ± 0.4 ^b^	nd
	Benzaldehyde	17.4 ± 0.1 ^b^	15.8 ± 0.3 ^a^	17.2 ± 0.4 ^b^	16.9 ± 0.3 ^b^	18.4 ± 0.2 ^c^
	Sub-total	58.0 ± 0.5 ^d^	35.6 ± 0.3 ^b^	39.7 ± 2 ^c^	33.2 ± 0.9 ^a^	36.1 ± 0.6 ^b^
Esters	Ethyl acetate	13.8 ± 0.2 ^a^	nd	70.6 ± 0.8 ^b^	nd	nd
	Octanoic acid ethyl ester	15.7 ± 0.2 ^a^	43.1 ± 0.3 ^d^	25.4 ± 0.4 ^b^	26.1 ± 0.5 ^b^	27.6 ± 0.3 ^c^
	cis-4-Hydroxy-3-methylundecanoic acid lactone	3.81 ± 0.05 ^a^	nd	nd	nd	nd
	Dibutyl phthalate	nd	7.03 ± 0.07 ^a^	12.4 ± 0.2 ^d^	9.59 ± 0.10 ^c^	7.29 ± 0.15 ^b^
	Decanoic acid ethyl ester	22.6 ± 0.3 ^d^	3.87 ± 0.05 ^b^	8.00 ± 0.08 ^d^	1.90 ± 0.02 ^a^	6.80 ± 0.07 ^c^
	4-Ethoxy-benzoic acid ethyl ester	21.9 ± 0.5 ^a^	nd	nd	nd	nd
	3-Hydroxy-butanoic acid methyl ester	nd	1.63 ± 0.06 ^a^	nd	nd	6.39 ± 0.03 b
	2-Butoxy-1-methyl-2-oxoethyl butanoic acid ester	nd	13.4 ± 0.2 ^b^	13.7 ± 0.3 ^b^	0.60 ± 0.01 ^a^	13.5 ± 0.4 ^b^
	Ethyl oxamate	nd	2.30 ± 0.06 ^c^	1.25 ± 0.03 ^a^	1.67 ± 0.06 ^b^	9.11 ± 0.08 ^d^
	δ-Decalactone	nd	nd	25.2 ± 0.4 ^a^	nd	nd
	Sub-total	78 ± 5 ^e^	72 ± 3 ^d^	93.0 ± 0.9 ^c^	39.8 ± 0.3 ^a^	70.8 ± 0.5 ^b^
Hydrocarbons	Undecane	nd	6.30 ± 0.05 ^c^	4.92 ± 0.03 ^b^	3.52 ± 0.08 ^a^	7.14 ± 0.09 ^d^
	Tetradecane	nd	nd	nd	37.2 ± 0.5 a	nd
	Tridecane	44.0 ± 0.3 ^d^	16.6 ± 0.5 ^bc^	2.08 ± 0.07 ^a^	15.7 ± 0.6 ^b^	17.6 ± 0.5 ^c^
	Dodecane	nd	nd	10.7 ± 0.1 ^b^	1.58 ± 0.04 ^a^	28.6 ± 0.3 ^c^
	5-Ethyldecane	nd	nd	nd	8.76 ± 0.1 ^b^	2.03 ± 0.06 ^a^
	3,3-Dimethyl-hexane	nd	6.53 ± 0.07 ^b^	nd	10.5 ± 0.1 ^c^	1.08 ± 0.02 ^a^
	2,5,9-Trimethyl-decane	4.64 ± 0.05 ^a^	5.38 ± 0.06 ^b^	nd	22.7 ± 0.3 ^d^	10.1 ± 0.1 ^c^
	Sub-total	9.04 ± 0.11 ^a^	34.8 ± 0.3 ^c^	17.7 ± 0.2 ^b^	99.9 ± 1.1 ^e^	66.6 ± 0.5 ^d^
Heterocyclic compounds	Indole	nd	11.9 ± 0.2 ^a^	12.4 ± 0.3 ^a^	12.4 ± 0.2 ^a^	14.1 ± 0.4 ^b^
	Maltol	1.20 ± 0.06 ^a^	1.07 ± 0.07 ^a^	1.13 ± 0.02 ^a^	12.9 ± 0.3 ^b^	12.6 ± 0.1 ^b^
	2-Pentyl-furan	4.26 ± 0.06 ^a^	nd	nd	nd	nd
	Furfural	7.02 ± 0.09 ^a^	nd	nd	nd	nd
	Sub-total	12.5 ± 0.4 ^a^	12.9 ± 0.2 ^a^	13.5 ± 0.3 ^d^	25.3 ± 0.4 ^b^	26.7 ± 0.5 ^c^
Ketones	Acetoin	139.6 ± 2 ^d^	60.8 ± 0.5 ^b^	85 ± 0.02 ^c^	55.8 ± 0.9 ^a^	53.4 ± 0.9 ^a^
	Sub-total	139.6 ± 2 ^d^	60.8 ± 0.5 ^b^	85 ± 0.02 ^c^	55.8 ± 0.9 ^a^	53.4 ± 0.9 ^a^
	Total	1667 ± 25 ^d^	1220 ± 18 ^b^	1371 ± 13 ^c^	1268 ± 11 ^a^	1332 ± 24 ^b^

Means ± standard deviation (*n* = 3); different small superscripts in the same row and column, respectively, indicate significant differences at *p* < 0.05. nd: Not detected.

**Table 5:** The effect of in situ-formed EPS on sensory evaluation of sourdough bread.

Samples	Color	Taste	Appearance	Overall Acceptance
CW	6.60 ± 0.18 ^a^	7.15 ± 0.25 ^c^	7.01 ± 0.12 ^b^	7.1 ± 0.01 ^ab^
CR	6.90 ± 0.14 ^ab^	6.81 ± 0.19 ^b^	6.53 ± 0.13 ^a^	6.8 ± 0.03 ^a^
WR	7.00 ± 0.25 ^b^	7.13 ± 0.16 ^c^	7.39 ± 0.01 ^c^	7.3 ± 0.05 ^b^
AWR	6.90 ± 0.11 ^ab^	6.16 ± 0.05 ^a^	6.91 ± 0.05 ^b^	7.0 ± 0.01 ^ab^
EWR	6.90 ± 0.13 ^ab^	7.01 ± 0.12 ^c^	7.0 ± 0.05 ^b^	7.1 ± 0.02 ^ab^

Means ± standard deviation (*n* = 3). Sensory evaluation was performed based on a 9-point hedonic scale from 1 ”extremely dislike” to 9 “like extremely”. Different small superscripts in the same column indicate significant differences at *p* < 0.05.

## Data Availability

The data presented in this study are available on request from the corresponding author.

## Supplementary Materials

The following supporting information can be downloaded at: https://www.mdpi.com/article/10.3390/foods12030605/s1, Table S1:Composition of controls and red bean sourdough bread.
