# Supplementary material for: Rheo-Fermentation Dough Properties, Bread-Making Quality and Aroma Characteristics of Red Bean (Vigna angularis) Sourdough Induced by LAB Weissella confusa QS813 Strain Fermentation
Source: Foods. 2023 Feb 1;12(3):605. doi: 10.3390/foods12030605 (PMC9913992; doi:10.3390/foods12030605)
Supplement: Supplementary file 1 [file foods-12-00605-s001.zip › foods-2122726-supplementary.pdf]

Table S1. Composition of controls and red bean sourdough bread.

Ingredient (s)	Sample				
	WR	EWR	CR	AWR	CW
Wheat flour (g)	90	89.81	90	90	100
Water (mL)	66	66	66	66 **	66
Red bean flour (g)	-	10	10	10	-
Red bean sourdough (g)	10	-	-	-	-
EPS (g)	-	0.19 *	-	-	-
Yeast (g)	2	2	2	2	2
Butter (g)	8	8	8	8	8
Sugar (g)	10	10	10	10	10
Salt (g)	1.5	1.5	1.5	1.5	1.5

WR: Red bean sourdough dough. EWR: Red bean flour dough, containing EPS extracted from red bean sourdough fermented by *W. confusa* QS813. CR: Red bean flour dough. AWR: Red kidney bean flour dough, chemically acidified to similar pH as WR. CW: Wheat flour dough. * EPS was extracted from red bean sourdough fermented by *W. confusa* QS813 as described by Tang et al. [20]. ** Chemically acidified using acetic acid and lactic acid at a molar ratio of 0.66:0.33.
